# Secondary Hyperparathyroidism and Cognitive Decline

**DOI:** 10.1007/s40472-023-00394-5

**Published:** 2023-03-15

**Authors:** Philip Crepeau, Tatiana Fedorova, Lilah F. Morris-Wiseman, Aarti Mathur

**Affiliations:** 1 Department of Surgery, Johns Hopkins University School of Medicine, 600 North Wolfe Street, Blalock 606, Baltimore, MD 21287, USA

**Keywords:** Secondary hyperparathyroidism, Cognitive decline, Dementia, Parathyroid hormone, End-stage kidney disease, Chronic kidney disease

## Abstract

**Purpose of Review:**

Secondary hyperparathyroidism (SHPT) likely contributes to the high prevalence of cognitive decline found among individuals with end-stage kidney disease (ESKD). Our objective is to critically evaluate the recent literature regarding the association between SHPT and cognitive decline and identify potential mechanisms.

**Recent Findings:**

Nine studies assessing the relationship between SHPT and cognition have been published in the last two decades, each showing that elevated parathyroid hormone (PTH) levels were associated with cognitive decline. One also found structural changes within the brain related to SHPT. Additionally, two found that SHPT treatment decreases the risk of cognitive decline in ESKD patients.

**Summary:**

SHPT is associated with cognitive impairment. However, the severity of SHPT associated with these changes and the specific cognitive domains affected remain unclear. Future studies are needed to focus on specific cognitive domains, the trajectory of cognitive decline, and optimal treatment strategies including the impact of kidney transplant and tertiary hyperparathyroidism.

## Introduction

Secondary hyperparathyroidism (SHPT), characterized by elevated parathyroid hormone (PTH) levels secondary to disruption in calcium and vitamin D homeostasis, is a normal physiologic response that occurs with progressive kidney disease [1]. PTH levels begin to rise beginning in stage 3 chronic kidney disease (CKD) such that SHPT is present in nearly all those with end-stage kidney disease (ESKD) at the time of dialysis initiation [2, 3]. However, the gradual rise in PTH levels eventually becomes pathologic; severely elevated PTH levels in SHPT are associated with up to a 24% increased risk of all-cause mortality [4-7]. Thus, current guidelines recommend optimizing PTH levels with medical treatment or surgical parathyroidectomy [8, 9].

In the past two decades, newer and less-recognized sequelae of elevated PTH have emerged, including impaired cognition. Among individuals with normal renal function, elevated PTH is associated with cognitive impairment and improves with treatment [10-16]. This has led some investigators to examine cognitive decline and dementia among ESKD patients who have a high incidence of cognitive impairment and significantly higher PTH levels compared to those with normal renal function.

Up to 70% of individuals with ESKD have cognitive impairment and up to 16% of older adults risk developing dementia at 5 years following dialysis initiation [17, 18, 19, 20]. Compared to individuals with normal renal function, those with ESKD have up to a threefold greater likelihood of cognitive impairment [21-28]. Cognitive impairment with and without functional dependence exists even among younger patients with ESKD [29]. The reasons for cognitive impairment among individuals with ESKD are likely multifactorial and include toxic uremia, polypharmacy, intermittent cerebral ischemia, anemia, and hyperparathyroidism [21, 30]. Cognition includes multiple domains including global cognitive function, executive function, memory, language, attention, motor skills, and visuospatial perception [31]. While intermittent cerebral ischemia during dialysis has been previously associated with decreased executive function in those with ESKD [32], it is unclear which domain of cognition is affected by hyperparathyroidism.

Furthermore, treatment of SHPT with phosphate binders, vitamin D analogs, calcimimetics, or parathyroidectomy is effective in lowering PTH levels. Hyperparathyroidism, therefore, represents a modifiable risk factor for cognitive impairment and dementia in SHPT patients although it is currently not included as a factor in clinical decision-making. Due to the paucity of data surrounding SHPT and cognition, it is difficult to determine whether cognitive decline in individuals with ESKD is due to SHPT or some other factors.

Therefore, the purpose of this review is to critically evaluate the recent literature regarding the association between SHPT and cognitive decline and identify possible mechanisms for this association.

## Association Between SHPT and Cognitive Decline

There have been 7 studies published between 2006 and 2020 from 6 different countries that have evaluated the association between SHPT and cognitive decline (Table 1). Of these, four were cross-sectional studies [33•, 34•, 35•, 36••], one was a case-control [37•], and two were systematic reviews [10, 11]. The study populations of the four cross-sectional studies included those with CKD and ESKD [33•, 34•, 35•, 36••], while the case-control study included only those with normal renal function [37•]. Sample sizes in these studies ranged from 40 to 256 patients.

PTH levels were measured as continuous variables in four studies [34•, 35•, 36••, 37•]. Conversely, one study treated PTH as a categorical variable and compared those with PTH levels between 150 and 300 mg/dL to those with PTH levels either higher or lower than this range [33•]. Only four cognitive domains were assessed: global cognitive function, executive function, memory, and language. Therefore, evidence is lacking for domains of attention, motor skills, and visuospatial perception.

### SHPT and Global Cognitive Function

Three studies assessed global cognitive function using either the Mini Mental State Exam (MMSE), the clock-drawing test, or the Montreal Cognitive Assessment (MoCA) [33•, 34•, 36••] (Table 2). Utilizing the MMSE, Leinau et al. found that among individuals with ESKD, PTH values greater or less than 150–300 mg/dL were associated with lower MMSE scores on regression models (*r* = − 0.158, *p* = 0.05), indicating worse global cognitive function [33•]. Kalaitzidis et al. further found that increasing PTH levels measured on a continuous model were associated with moderate or severe cognitive impairment on the MMSE (adjusted OR = 1.01, 95% CI: 1.00–1.01) and the clock-drawing test (aOR = 1.92, 95% CI: 1.23–2.99) after adjusting for the following confounders: CKD stage, age, presence of diabetes, and diastolic blood pressure [34•]. Gong et al. found that average MoCA scores among individuals with ESKD were lower in those with SHPT compared to those without SHPT (22.92 ± 2.22 vs. 24.96 ± 1.59, *p* = 0.01), although the authors did not specify the criteria used to define SHPT [36••].

In total, these three studies including 412 total patients found that SHPT was associated with worse global cognitive function. However, only Kalaitzidis adjusted for confounders in their analysis [34•], making it difficult to ascertain the true association between SHPT and global cognitive function. Furthermore, there was significant heterogeneity in the methods used to assess PTH levels among these studies: Leinau measured PTH levels categorically, Kalaitzidis measured them continually, and Gong did not define the PTH level used to identify those with SHPT. Therefore, the PTH threshold at which SHPT may lead to cognitive impairment remains unclear. The lack of inclusion of confounders and consistent PTH thresholds limit the ability to incorporate these findings into clinical practice.

### SHPT and Executive Function

Four studies assessed executive function using multiple different tests [33•, 34•, 35•, 37•] (Table 2). Jorde et al. utilized six tests including the Trail Making Test parts A and B, the Stoop Color-Word Tests parts 1–3 (parts 1 and 2 scored together), the Digit Symbol test, and the California Computerized Assessment Package [37•]; Leinau utilized the 25-Item Executive Interview (EXIT-25) [33•]; Kalaitzidis utilized the Instrumental Activity of Daily Living (IADL) [34•]; and Puy et al. utilized six tests including the Trail Making Test parts A and B, the Stroop Category Fluency and Letter Fluency tests, the Wechsler Adult Intelligence Scale Digit Symbol Substitution test, and the Behavioral Dysexecutive Syndrome Inventory [35•]. The findings from these studies were mixed. Among ESKD patients, Leinau found that PTH levels greater or less than 150–300 pg/dL correlated with higher EXIT-25 scores (*r* = 0.185, *p* = 0.02), indicating worse executive function in these individuals [33•]. Puy calculated a cognitive summary score for patients with CKD based on the mean scores of tests measuring cognition in various domains, including executive function [35•]. While the authors found that increasing PTH levels on a linear regression model were associated with cognitive summary scores in the 5th percentile (*E* = − 1.655 × 10^−3^, *p* = 0.007), they did not evaluate a specific association between PTH levels and executive function [35•].

On the other hand, Jorde found that those with SHPT, defined as serum calcium < 9.62 mg/dL and PTH > 60.8 pg/mL, performed worse on only one of six tests of executive function (Stroop Color-Word test parts 1 and 2) when compared to those without SHPT (55.1 ± 10.9 vs. 49.0 ± 9.4, *p* < 0.05) after adjusting for age, sex, BMI, education, and health score [37•]. Additionally, Kalaitzidis found that increasing PTH levels measured on a continuous model were not associated with moderate or severe cognitive impairment as measured by the IADL (OR = 1.01, 95% CI: 0.99–1.05).

In summary, the association between SHPT and executive function has yet to be delineated. Two studies consisting of a total of 146 patients found that SHPT may be associated with worse executive dysfunction, although the studies varied in both the methods used to measure executive function as well as the statistical analyses used to calculate this association [33•, 35•]. In contrast, two studies consisting of a total of 340 total patients found a mixed association [37•] or no association at all [34•] between SHPT and worse executive function. Further studies are needed to identify the most appropriate screening test for executive function and determine whether SHPT is associated with worse executive function.

### SHPT and Memory

Two studies assessed memory using various methods [35•, 37•] (Table 2). Jorde utilized the Wechsler Memory Scale–Revised test (Digit Span Forward, Digit Span Backwards, Verbal Recall, and Visual Recall tests), the Seashore Rhythm Test, and the California Verbal Learning Test [37•]. Puy utilized three tests for memory, including the Freed and Cued Selective Recall Test, the Baddeley Door test, and the Rey-Osterrieth Complex Figure Test [35•]. Results from these studies are also mixed. Jorde found that those with SHPT, defined as serum calcium < 9.62 mg/dL and PTH > 60.8 pg/mL, performed worse on only one of six memory tests (Digit Span Forward Test) compared to those without SHPT (5.0 ± 0.8 vs. 5.8 ± 1.1, *p* < 0.05) after adjusting for age, sex, BMI, education, and health score [37•]. Puy incorporated tests of memory into a cognitive summary score and found that increasing PTH levels on a linear regression model were associated with cognitive summary scores in the 5th percentile (*E* = − 1.655 × 10^−3^, *p* = 0.007), although the authors did not indicate the specific association between PTH levels and memory [35•].

Thus, these two studies consisting of 124 patients show some evidence that SHPT may be associated with poorer memory although the exact nature of this association is unclear. A total of nine tests of memory were used between these two studies, each with mixed results, which limit the interpretation of these findings. Further studies with a uniform method of testing memory would aid in further evaluating the association between SHPT and this domain of cognitive function.

### SHPT and Language

Two studies assessed language skills [35•, 37•] (Table 2). Puy incorporated scores from the Boston Naming Test into a cognitive summary score and found that increasing PTH levels on a linear regression model were associated with cognitive summary scores in the 5th percentile (*E* = −1.655 × 10^−3^, *p* = 0.007), although the authors did not indicate the specific association between PTH levels and language [35•]. Additionally, Jorde found that those with SHPT, defined as serum calcium < 9.62 mg/dL and PTH > 60.8 pg/mL, performed worse on the Controlled Word Association Test compared to those without SHPT (28.4 ± 12.9 vs. 37.6 ± 14.1, *p* < 0.01) after adjusting for age, sex, BMI, education, and health score [37•].

These two studies including 124 patients suggest that SHPT may be associated with language impairment. However, neither study adjusts for potential confounders in their analysis, making it difficult to determine whether this association exists. Additionally, these studies are limited by their small sample sizes. As such, further research is warranted to better understand the association between SHPT and language impairment.

### Limitations

While the above studies provide evidence that SHPT may affect various domains of cognition, there are several limitations to consider when interpreting these data. First, there is significant heterogeneity in the specific assessments utilized and cognitive domains measured, with lack of data regarding the domains of attention, motor skills, and visuospatial perception. It is therefore difficult to identify patterns of association between SHPT and impairment of specific cognitive domains. Because cognitive decline in those with ESKD is likely multifactorial, understanding the specific domains of cognitive decline that are impacted by SHPT, as well as the particular screening test that can identify this decline, may aid in recognizing when cognitive decline in those with ESKD may be attributed to SHPT. This would provide clinically meaningful information and alert providers as to whether SHPT needs to be treated more aggressively.

The above studies are also limited in their statistical approach, as only two of the five studies adjusted for potential confounders [34•, 37•]. Although both of these studies adjusted for confounders such as age, sex, BMI, and level of education, neither adjusted for other known causes of cognitive decline among ESKD patients including blood urea nitrogen levels, hematocrit, number of medications taken, number of years on dialysis, or dialysis modality, each of which may affect the association between SHPT and cognitive decline. The lack of consistent adjustment for confounders across each study makes it difficult to ascertain the true nature of the association between SHPT and cognitive decline.

The two systematic reviews each came to similar conclusions: While some evidence exists to support an association between SHPT and cognition, the findings are mixed and the quality of the data to support these findings are low to moderate [10, 11]. However, these reviews contained heterogeneous study populations that included multiple studies of patients with normal renal function, thus limiting the generalizability of these findings to ESKD patients.

Finally, only two studies used specific PTH levels to assess the impact of SHPT on cognition [33•, 37•]. As such, the PTH threshold that is associated with cognitive decline is unclear, and the trajectory of cognitive decline is unknown. Such a threshold would give providers a target for PTH levels to improve or prevent cognitive decline. The existing studies are limited in their scope as well, only focusing on patients with hyperparathyroidism while on dialysis. There is a paucity of data regarding post-transplant hyperparathyroidism and cognitive trajectories. Further studies are therefore needed to understand optimal PTH thresholds both pre-transplant and post-transplant in order to better aid in clinical decision-making.

## Mechanism of Cognitive Decline in SHPT

While the exact mechanism of cognitive decline in patients with SHPT is likely multifactorial, several studies attempted to identify the role of PTH in cognitive impairment. PTH may interact with the brain directly and cause functional and anatomical changes. Gong found that among individuals with ESKD, those with SHPT had significantly increased gray matter volume in the bilateral caudate and bilateral thalamus compared to those without SHPT [36••]. These anatomical changes correlated with poorer performance on the MoCA. PTH may cause these changes directly by binding to receptors such as PTH2, which are abundant in the brain and other neural tissues [38]. PTH may also cause functional changes within the brain. Intracerebral administration of PTH has been shown to affect dopamine metabolism and prolactin release which in turn and has effects on memory, nociperception, and serum calcium [38, 39].

In addition to its direct effects on neural tissue, PTH may also mediate vascular changes in the brain. Hyperparathyroidism in those with CKD can lead to increased calcium-phosphate product and vascular calcification development [40]. Thus, elevated PTH may predispose affected individuals to cerebrovascular disease. Hagström et al. found that higher PTH levels were associated with a greater risk of vascular dementia [41]. On imaging, elevated PTH also correlates with increased white matter hyperintensities associated with small vessel cerebral disease [41]. Therefore, cognitive dysfunction in those with SHPT may be the result of subclinical cerebrovascular disease or silent infarcts.

Elevated PTH may also cause cognitive dysfunction indirectly by interfering with neurotransmitter metabolism. Smogorzewski et al. found increased acetylcholine release from neurons in rat models of CKD compared to rats with normal renal function; increased acetylcholine release correlated with behavioral and motor changes [42]. The authors found that acetylcholine levels normalized following parathyroidectomy, suggesting an interaction between PTH and the neuronal synaptosome. While these data provide evidence for an association between elevated PTH levels and cognitive decline, further studies are needed to better elucidate the exact mechanism of cognitive decline in those with SHPT.

## SHPT Treatment and Cognitive Decline

Treatment for SHPT includes medical treatment, surgical parathyroidectomy, or waiting until kidney transplant to reverse the metabolic abnormalities giving rise to SHPT. Two studies have assessed the impact that treatment of SHPT has on cognitive decline (Table 3). In a case-control study of 62 dialysis patients, Chou et al. [43••] compared 39 individuals who underwent parathyroidectomy for symptomatic SHPT with a control group of 23 SHPT patients who did not undergo surgery. Symptomatic SHPT was defined as bone pain, pruritis, general weakness, and insomnia. MMSE and Clinical Dementia Rating (CDR) tests were administered prior to and at 16 weeks following surgery or at a 16-week interval for the non-surgical group. Baseline scores were similar between the two groups. MMSE scores improved significantly after parathyroidectomy (25 ± 5 to 26 ± 5, *p* < 0.001). Additionally, CDR scores improved in the parathyroidectomy group. In contrast, MMSE and CDR scores in the control group remained the same during the 16-week interval suggesting that parathyroidectomy may slow or even reverse the trajectory of cognitive decline in those with SHPT [43••].

Treatment of SHPT may decrease the risk of dementia as well. In a retrospective claim-based study of 189,433 older adults (age ≥ 66) who were initiating dialysis, 65% were treated for SHPT either medically (using vitamin D analogs, phosphate binders, or calcimimetics) or surgically (parathyroidectomy) during the 10 years of follow-up [44••]. Compared with the untreated group, the risk of developing incident dementia was 42% lower in the treated group and the risk of developing Alzheimer’s disease was 33% lower in the treatment group. This risk did not vary by treatment type, with medically and surgically treated patients having similar incident rates of dementia.

## Impact of Kidney Transplant on Cognition

Cognitive decline presents several challenges for kidney transplant (KT) recipients. In patients with ESKD, cognitive impairment decreases the likelihood of being listed for transplant by 25% [45]. Once listed, patients with cognitive impairment and without diabetes have a 2.47 times greater risk of wait-list mortality [45]. Cognitive impairment also leads to significantly diminished health literacy among transplant candidates [46]. Following transplant, KT recipients must follow complex regimens of immunosuppressive medications and require close follow-up with multiple clinic visits, and lack of adherence to these measures is a major contributor to graft loss and mortality [47, 48]. When compared to KT recipients with normal cognition, those with severe cognitive impairment have up to a 5.57 times greater risk of all-cause graft loss by 5 years following KT [49].

There is some evidence to suggest that cognitive decline may improve following KT. Several studies have shown that global cognitive function, motor skills, and memory may improve following KT, although these studies were limited by small sample sizes and short follow-up times [50-53]. In a larger study of 665 patients undergoing KT, global cognitive function improved significantly over the first 3 months following KT and was sustained for up to 4 years [54]. However, not all aspects of cognition improve following KT. A study of 894 KT recipients found that executive function declined steadily up to 2 years following transplant [55]. Despite any improvements in cognition that may result from transplant, KT recipients still have worse cognition compared to age-matched, sex-matched, and education-matched controls without ESKD, suggesting that transplantation does not completely reverse ESKD-associated cognitive decline [52, 56, 57]. Additionally, a recent meta-analysis of 1118 KT recipients found that cognitive domains including executive function, language, and attention did not improve following transplantation and were not superior to ESKD patients who had not undergone transplant [58].

It is uncertain whether any changes in cognition following KT are related to improvement of SHPT. ESKD-related hyperparathyroidism does not always resolve following KT, and PTH levels may remain persistently elevated in up to 60% of patients at 1 year following transplantation [59-62]. Of those with hyperparathyroidism at 1 year following transplant, roughly 35% have concomitant hypercalcemia, known as tertiary hyperparathyroidism (THPT) [63]. To our knowledge, there have not been any studies evaluating the role of THPT and its impact on post-KT cognitive trajectory. Additionally, the studies assessing the impact of KT on cognitive function do not account for PTH levels, and thus the association between post-transplant SHPT or THPT and cognitive decline is unclear. Further studies are warranted in order to determine whether changes in PTH levels following KT are associated with improvements in cognitive function.

## Conclusion

In reviewing data on the association between SHPT and cognition, while a significant association between SHPT and cognitive decline was identified in each study, the literature is heterogeneous with regard to specific cognitive domains that are affected, thus limiting the inferences and generalizability of the data. PTH thresholds for optimal cognition are unclear, which limits clinical decision-making. Future studies are needed to better understand the specific cognitive domains affected by SHPT and the PTH levels that achieve optimal cognition. Additionally, further prospective studies are warranted in order to determine how treatment impacts these cognitive trajectories.

## Figures and Tables

**Table 1:** Summary of recent studies assessing the association between SHPT and cognitive decline

Author	Year	Country	Study Design	Study population	Sample Size	Covariates Adjusted	Cognitive Domain Assessed			
							Global	Executive Function	Memory	Language
Jorde (37)	2006	Norway	CC	SHPT	84	Yes		Mixed	Mixed	Y
Leinau (33)	2009	USA	CS	ESKD	106	No	Y	Y		
Kalaitzidis (34)	2013	Greece	CS	CKD 1-5	256	Yes	Y	N		
Lourida (10)	2015	UK	SR	Multiple	27^a^	n/a	Y	Mixed	Y	
Puy (35)	2018	France	CS	CKD 2-5	40	No		Y	Y	Y
Gong (36)	2020	China	CS	ESKD	50	No	Y			
Jiang (11)	2020	China	SR	Multiple	10^a^	n/a	Y	Y		

Shaded area = not assessed

Abbreviations: *CC* case-control; *CS* cross-sectional; *SR* systematic review; *ESKD* end-stage kidney disease; *SHPT* secondary hyperparathyroidism; *CKD* chronic kidney disease; *Y* yes, association; *N* no association

a Refers to number of studies included in the systematic review

**Table 2:** Tests administered in recent studies to assess cognitive domains

Cognitive domain	Author	Cognitive test
Global cognitive function	Leinau (33)	MMSE
	Kalaitzidis (34)	MMSE and clock-drawing test
	Gong (36)	MoCA
Executive function	Jorde (37)	Trail Making Tests parts A and B
		Stroop Color-Word Tests parts 1–3
		Digit Symbol Test
		California Computerized Assessment Package
	Leinau (33)	EXIT-25
	Kalaitzidis (34)	IADL
	Puy (35)	Trail Making Tests parts A and B
		Stroop Category and Letter Fluency Test
		Wechsler Adult Intelligence Scale Digit Symbol Substitution Test
		Behavioral Dysexecutive Syndrome Inventory
Memory	Jorde (37)	Digit Span Forward and Backwards
		Verbal and Visual Recall Test
		Seashore Rhythm Test
		California Verbal Learning Test
	Puy (35)	Freed and Cued Selective Recall Test
		Baddeley Door Test
		Rey-Osterrieth Complex Figure Test
Language	Jorde (37)	Controlled Word Association Test
	Puy (35)	Boston Naming Test

Abbreviations: *MMSE* Mini Mental State Exam; *MoCA* Montreal Cognitive Assessment; *EXIT-25* 25-Item Executive Interview; *IADL* Instrumental Activity of Daily Living

**Table 3:** Studies assessing the impact of SHPT treatment on cognitive decline or incident dementia

Author	Year	Country	Study design	Study population	Sample size	Outcome	Exposure	Results
Chou (43)	2008	Taiwan	CC	SHPT	63	Change in MMSE score	PTX	Mean MMSE scores improved 16 weeks after PTX (25 ± 5 to 26 ± 5, *p* < 0.001)
Mathur (44)	2022	USA	CS	ESKD	189,433	Incidence of dementia	Treatment of SHPT	Treating SHPT decreases the risk of dementia (aHR = 0.58, 95% CI: 0.56–0.59)

Abbreviations: *CC* case-control; *CS* cross-sectional; *ESKD* end-stage kidney disease; *MMSE* Mini Mental State Exam; *SHPT* secondary hyperparathyroidism; *PTX* parathyroidectomy; *aHR* adjusted hazard ratio; *CI* confidence interval

## Data Availability

The datasets used and/or analyzed during the current study are available from the corresponding author on reasonable request.
